# Consistency of drug-resistant mutations in plasma and peripheral blood mononuclear cells of patients with treatment-naïve and treatment-experienced HIV-1 infection

**DOI:** 10.3389/fcimb.2023.1249837

**Published:** 2023-12-19

**Authors:** Jie Ma, Zhaoyun Chen, Chaohong Fu, Shuguang Wei, Jinjin Liu, Xuan Yang, Xuhui Chen, Qingxia Zhao, Yan Sun, Yuqi Huo

**Affiliations:** Center for Translational Medicine, The Sixth People’s Hospital of Zhengzhou, Zhengzhou, China

**Keywords:** genotypic drug resistance, drug-resistant mutations, proviral DNA, HIV RNA, peripheral blood mononuclear cells

## Abstract

**Introduction:**

Genotypic drug resistance testing is cursrently recommended by the World Health Organization for all patients infected with human immunodeficiency virus type 1 (HIV-1) undergoing care or switching regimes due to failure with previous antiretroviral therapy (ART). Patients with human immunodeficiency virus/acquired immunodeficiency syndrome (HIV/AIDS) who meet the criteria for free testing for genotypic drug resistance due to poor adherence in Henan Province may resume their previous regimens before resampling. Therefore, resistance testing based on plasma RNA can fail in a proportion of patients. Resistance testing based on peripheral blood mononuclear cells (PBMCs) is an alternative option. In this study, we investigated the differences in drug-resistant mutations (DRMs) between plasma HIV RNA and proviral DNA in treatment-experienced and treatment-naïve patients.

**Methods:**

Matched plasma RNA and proviral DNA samples of 66 HIV-1 infected treatment-naïve and 78 treatment-experienced patients were selected for DRM analysis and comparison.

**Results:**

DRMs were detected in 27.3% (18/66) of treatment-naïve and 80.8% (63/78) of treatment-experienced samples. Resistance to at least one drug was detected based on analysis of plasma RNA and proviral DNA in 7.6% (5/66) and 9.1% (6/66) of treatment-naïve patients and in 79.5% (62/78) and 78.2% (61/78) of treatment-experienced patients, respectively. Furthermore, 61/66 (92.4%) of treatment-naïve patients showed concordant RNA and DNA drug resistance. When drug resistance was defined as intermediate and high, the concordance of drug resistance profiles of paired RNA and proviral DNA samples derived from treatment-naïve patients were up to 97.0% compared with only 80.8% (63/78) in treatment-experienced patients.

**Discussion:**

Our data indicate that drug resistance testing based on plasma RNA or proviral DNA might be interchangeable in treatment-naïve patients, whereas plasma RNA-based testing remains the best choice for drug resistance analysis in patients with ART failure in clinical practice.

## Introduction

Data from the Chinese Center for Disease Control and Prevention (China CDC) showed that by the end of 2020, more than one million people were living with human immunodeficiency virus type 1 (HIV-1) in China. Human immunodeficiency virus/acquired immunodeficiency syndrome (HIV/AIDS) has been reported in all 31 provinces, including autonomous regions and municipalities, in China. China’s Free Antiretroviral Therapy (ART) Program officially launched in 2003 provides highly active antiretroviral therapy (HAART) or combination antiretroviral therapy (cART) for HIV-1-infected individuals to suppress the HIV-1 virus, and prevent the development of AIDS-associated opportunistic diseases and HIV-1 transmission (Cohen et al., 2016; Cao et al., 2020). During the first ten years of program implementation, the mortality rate associated with HIV/AIDS declined significantly (Cao et al., 2020). However, HIV/AIDS still represents a serious threat to public health because of ART failure due to poor compliance, host factors, and pharmacokinetic drug interactions, especially drug resistance (Chew et al., 2005; Phillips et al., 2005). Results from many laboratories have confirmed that drug-resistant mutations (DRMs) are frequently associated with the failure of ART (Phillips et al., 2005; von Wyl et al., 2007; Kiptoo et al., 2013). It has been reported that nearly half of ART failures in China are associated with DRMs (Wu et al., 2014; Luo et al., 2019).

Drug-resistant HIV-1 strains can be transmitted to newly infected individuals, which is a huge challenge for HIV elimination. The prevalence of transmitted drug resistance (TDR) in the USA and Europe ranged from 8% to 17.5% (Huang et al., 2021). Uneven geographic distribution of TDR was reported in China, with a prevalence ranging from 3.6% to 11.1% (Cao et al., 2020). The percentage of patients with one or more major DRMs was 6.4% in treatment-naïve individuals living in Henan Province (Yang et al., 2020). Primary infection with HIV TDR in addition to ART failure limits the treatment options available for infected patients (Gupta et al., 2018; Chimukangara et al., 2019; Yuan et al., 2021). Timely diagnosis and management of drug resistance in patients with HIV-1 failing ART have the potential to improve treatment outcomes (Gupta-Wright et al., 2020). Drug resistance testing is recommended for individuals with primary HIV infection to guide the selection of the initial ART regimen, and for treatment-experienced patients with virological failure to assist with the selection of active drugs when changing ART regimens (Vandamme et al., 2011). While plasma RNA is recommended for drug resistance analysis, previous studies have suggested the use of proviral DNA with plasma RNA-based resistance testing to increase the sensitivity of DRM detection (Vicenti et al., 2007; Lübke et al., 2015). Further, a certain proportion of patients with HIV/AIDS who meet the criteria for free genotypic drug resistance testing (viral load [VL] > 1,000 copies/mL) due to poor adherence in Henan Province may resume regular medication as requested by their physicians before subsequent blood sampling, which inevitably leads to failure of resistance testing based on plasma RNA, thereby making resistance testing based on peripheral blood mononuclear cells (PBMCs) an alternative option. However, results from paired plasma RNA- and proviral DNA-based resistance testing of treatment-naïve patients are conflicting, with some studies demonstrating discordance between the two compartments (Sarmati et al., 2003; Ellis et al., 2004; Chew et al., 2005; Bon et al., 2007; Parisi et al., 2007; Vicenti et al., 2007; Saracino et al., 2008), while others report contrary findings (Paolucci et al., 2001; Vicenti et al., 2007; Derache et al., 2015). Moreover, limited data are available to determine whether DRMs occur consistently in plasma RNA and proviral DNA of patients with ART failure.

In this study, we investigated the differences in DRMs between plasma HIV RNA and HIV proviral DNA of treatment-experienced and treatment-naïve patients.

## Materials and methods

### Ethical statement

The study was approved by the Ethics Committee of the Sixth People’s Hospital of Zhengzhou and written informed consent was obtained from all patients.

### Participants

Patients admitted to The Sixth People’s Hospital of Zhengzhou in Henan Province, China, with no history of ART or who experienced ART failure were enrolled in this study from Oct 2019 to Dec 2021. ART failure was defined as a single VL ≥ 1000 copies/mL. Data regarding the patients’ general condition, transmission route, HIV-1 RNA VL, CD4+ T cell count, and history of ART were collected.

### Sample collection and processing

Whole blood samples were collected from HIV-1-infected patients and used to isolate PBMC using Ficoll density gradient centrifugation. The plasma in the upper layer of the gradient was also collected. Viral RNA in plasma samples was extracted using a Virus RNA Extraction Kit (Zhijiang, Shanghai, China). Total DNA in PBMCs was extracted using a Blood Genomic DNA Mini Kit (CoWin Biosciences, Jiangsu, China). The HIV-1 RNA VL in the plasma samples was determined using a HIV-1 RNA Quantitative Diagnostic Kit (Northeast Pharmaceutical Group, Shenyang, China).

### Genotypic drug resistance testing

For plasma RNA, HIV-1 partial *pol* gene sequence was first reverse-transcribed and then amplified as described previously (Yang et al., 2020). Briefly, the target sequence (approximately 1,300 base pairs) was reverse transcribed with the primer R5073 using a RevertAid First Strand cDNA Synthesis Kit (Thermo Fisher Scientific, MA, USA), and then amplified by nested polymerase chain reaction (PCR) using LA Taq™ Version 2.0 (Takara, Dalian, China) with primer PRO-F1 and RT-R1 for first-round PCR and primer PRO-F2 and RT-R2 for second-round PCR. The first-round amplification conditions were used as follows: 95°C for 3 min, followed by 30 cycles of 95°C for 10 s, 55°C for 30 s, and 72°C for 2 min, and a final extension at 72°C for 10 min. The second-round amplification conditions were used as follows: 95°C for 3 min, followed by 35 cycles of 95°C for 10 s, 55°C for 30 s, and 72°C for 90 s, and a final extension at 72°C for 10 min. For HIV-1 proviral DNA, the extracted DNA was directly used as a template for the amplification of the partial *pol* gene sequence and the same amplification strategy was used except for the omission of the reverse transcription. The primers used in this study are listed in **Supplementary Table 1**. For analysis of DRMs and drug resistance profile, sequences were submitted to the regularly updated Stanford HIV-1 drug resistance database (https://hivdb.stanford.edu/).

### Phylogenetic analysis

All paired sequences derived from treatment-naïve and treatment-experienced patients were aligned using Clustal W method as incorporated in Lasergene software and a phylogenetic tree was constructed using Molecular Evolutionary Genetic Analysis (MEGA) software program (version X) based on neighbor-joining method and Tamura-Nei model with 1000 bootstrap replicates (Yang et al., 2020).

### Data availability

Sequencing results have been submitted to the NCBI database. The DNA and RNA accession numbers of isolates from treatment-experienced patients are OP329553-OP329630 and OP329631-OP329708, respectively. The DNA and RNA accession numbers of isolates from treatment-naïve patients are OP329421-OP329486 and OP329487-OP329552, respectively.

### Statistical analysis

Statistical analysis was carried out using the SPSS statistics program (version 20) for Windows (SPSS, Inc., US). Continuous variables were expressed as mean ± standard deviation if normal or median with interquantile range (first to third quartile). Categorical variables were expressed as numbers or percentages. The differences between or among groups were analyzed via Student’s t-test or Chi-squared test or Fisher’s exact test. A P < 0.05 was considered statistically significant.

## Results

### Patients’ characteristics

A total of 148 HIV-1 infected individuals were included, among which 66 were classified as treatment-naïve and 82 as treatment-experienced patients. The partial *pol* gene sequence failed to amplify in 4 treatment-experienced patients and the data of 144 patents were thus included in the subsequent analysis. The median age of the included patients was 43 years, and males accounted for 79.2%. 95.8% of the patients came from Henan Province. Among these patients, heterosexual activity (HSX) and men having sex with men (MSM) were the two major transmission routes, accounting for 33.3% and 27.8%, respectively. The mean plasma RNA VL was 4.78 log_10_ copies/mL, and 44.4% of the patients were infected with subtype B viruses. Detailed characteristics of the included patients are listed in **Table 1**.

**Table 1:** Patients’s characteristics.

Characteristic	Total (n = 144)	Treatment-naïve (n = 66)	Treatment-experienced (n = 78)	P
Age (years)	42.6 ± 14.4	41.9 ± 13.5	43.2 ± 15.2	
sex				
Male-no.(%)	114 (79.2%)	58 (87.9%)	56 (71.8%)	
Female-no.(%)	30 (20.8%)	8 (12.1%)	22 (28.2%)	
Geographic location of subjects				
Henan-no.(%)	138 (95.8%)	61 (92.4%)	77 (98.7%)	
Other-no.(%)	6 (4.2%)	5 (7.6%)	1 (1.3%)	
Risk factor for HIV				
MSM-no.(%)	40 (27.8%)	24 (36.4%)	16 (20.5%)	
HSX-no.(%)	48 (33.3%)	24 (36.4%)	24 (30.8%)	
MTCT-no.(%)	7 (4.9%)	0 (0)	7 (9.0%)	
PL-no.(%)	12 (8.3%)	0 (0)	12 (15.4%)	
OTH-no.(%)	37 (25.7%)	18 (27.3%)	19 (24.4%)	
CD4^+^ T cell count (cells/μl)				0.435
< 200	107 (74.3%)	47 (71.2%)	60 (76.9%)	
≥200	37 (25.7%)	19 (28.8%)	18 (23.1%)	
HIV RNA Baseline viral load log (copies/ml)				0.029
mean ± SD	4.78± 0.86	4.95 ± 0.83	4.63 ± 0.86	
HIV-1 subtype				0.001
B-no.(%)	64 (44.4%)	19 (28.8%)	45 (57.7%)	
Non-B-no.(%)	80 (55.6%)	47 (71.2%)	33 (42.3%)	

MSM, men who have sex with men; HSX, heterosexual orientation; PL, plasmapheresis; MTCT, mother-to-child transmition; OTH, others, including blood transfusion and patients whose risk factors were unknown or patients who did not provide information.

### Treatment regimens

The ART regimens of three of the 78 treatment-experienced patients were unknown due to incomplete treatment records, while 39 patients reported switching of ART regimens. The duration of treatment ranged from 3 months to more than 20 years. Nucleoside reverse transcriptase inhibitors (NRTIs) were included in the treatment regimens of all patients, while non-nucleoside reverse transcriptase inhibitors (NNRTIs) were used for 61 patients, and protease inhibitors (PIs) for 24 patients (**Supplementary Table 2**).

### Higher consistency of predicted DRM profiles between plasma RNA and PBMC DNA in treatment-naïve patients

Partial HIV-1 *pol* gene sequence, which harbors the protease gene and part of the reverse transcriptase gene in matched plasma RNA and PBMC DNA, was successfully amplified in 144 patients, which included 66 treatment-naïve patients. DRMs were detected in 27.3% (18/66) of treatment-naïve patients. When categorized by sample type, DRMs were detected in 14 plasma RNA samples and 17 PBMC DNA samples. The rates of DRM detection in plasma RNA and PBMC DNA of patients not exposed to NRTIs, NNRTIs, and PIs were 1.5% (1/66), 18.2% (12/66), and 4.5% (3/66), and 1.5% (1/66), 22.7% (15/66), and 4.5% (3/66), respectively (**Table 2**). DRMs associated with plasma RNA and PBMC DNA of twelve patients were similar and led to resistance to NNRTIs in 1 patient and PIs in 2 patients. Six patients’ RNA and DNA samples showed discrepancies in DRMs, with higher numbers of DRMs detected in plasma RNA of 2 patients (2 vs. 1, and 1 vs. 0, respectively) and higher numbers of DRMs in PBMC DNA of 4 patients (1 vs. 0, 1 vs. 0, 1 vs. 0, and 1 vs. 0, respectively). Of the six patients with discrepancies in DRMs between plasma RNA and PBMC DNA, additional DRMs led to resistance to NNRTIs in 5 patients (**Table 3**).

**Table 2:** The percentage of patients with DRMs.

Drug category	Treatment-naïve patients (n = 66)		Treatment-experienced patients (n = 78)	
	RNA	DNA	RNA	DNA
NRTIs	1 (1.5%)	1 (1.5%)	58 (74.4%)	57 (73.1%)
NNRTIs	12 (18.2%)	15(22.7%)	60 (76.9%)	60 (76.9%)
PIs	3 (4.5%)	3 (4.5%)	8 (10.3%)	8 (10.3%)

NRTIs, Nucleoside reverse transcriptase inhibitors; NNRTIs, non-nucleoside reverse transcriptase inhibitors; PIs, protease inhibitors

**Table 3:** Distribution of major DRMs in treatment-naïve patients with discrepancies in DRMs between plasma RNA and PBMC DNA and drug resistance analysis.

Sample code	Subtype	RT		PR	NNRTI				
		NRTI	NNRTI	PI	DOR	EFV	ETR	NVP	RPV
HIVHN2020-SF728RNA	CRF01_AE	None	None	None	**S**	**S**	**S**	**S**	**S**
HIVHN2020-SF728DNA		None	**M230I**	None	**L**	**L**	**L**	**I**	**I**
HIVHN2020-SF832RNA	CRF07_BC	None	None	None	**S**	**S**	S	**S**	S
HIVHN2020-SF832DNA		None	**V106VM**	None	**I**	**H**	S	**H**	S
HIVHN2020-SF590RNA	B	None	None	None	S	S	S	S	S
HIVHN2020-SF590DNA		None	**V106VI**	None	S	S	S	S	S
HIVHN2020-SF1366RNA	CRF01_AE	None	**V106VI**, V179D	None	S	S	**L**	**L**	**L**
HIVHN2020-SF1366DNA		None	V179D	None	S	S	**S**	**S**	**S**
HIVHN2020-new2RNA	CRF01_AE	None	**E138EG**	None	S	S	S	S	**L**
HIVHN2020-new2DNA		None	None	None	S	S	S	S	**S**
HIVHN2020-new6RNA	CRF08_BC	None	None	None	S	S	S	S	**S**
HIVHN2020-new6DNA		None	**E138A**	None	S	S	S	S	**L**

Discordant DRMs between plasma RNA and PBMC DNA were shown in bold.

H, high resistant; I, intermediate resistant; L, low-level resistant; S, sensitive or potentially resistant.

DOR, doravirine; EFV, efavirenz; ETR, etravirine; NVP, nevirapine; RPV, rilpivirine.

Discordant DRMs between plasma RNA and PBMC DNA are shown in bold.

### Reduced consistency of predicted DRM profiles between plasma RNA and PBMC DNA in treatment-experienced patients

DRMs were detected in 80.8% (63/78) of treatment-experienced patients. When categorized by sample type, DRMs were detected in 63 plasma RNA and 63 PBMC DNA samples. DRMs in plasma RNA and PBMC DNA of 42 patients were similar and associated with resistance to NNRTIs in 41 patients, NRTIs in 38 patients, and PIs in 4 patients. The rates of DRMs in plasma RNA and PBMC DNA against NRTIs, NNRTIs, and PIs were 74.4% (58/78), 76.9% (60/78), and 10.3% (8/78), and 73.1% (57/78), 76.9% (60/78), and 10.3% (8/78), respectively (**Table 2**). 21 patients showed discrepancies in DRMs between RNA and DNA samples, with higher numbers of DRMs found in the plasma RNA of 10 patients and higher numbers of DRMs in PBMC DNA of 11 patients. Of the 21 patients with discrepancies in DRMs between plasma RNA and PBMC DNA, additional DRMs were associated with resistance to NRTIs in 9 patients, NNRTIs in 10 patients, and PIs in 4 patients (**Tables 4**–**7**). Detailed data pertaining to drug resistance in patients carrying varied DRMs in plasma RNA and PBMC DNA are listed in **Tables 6**, **7**, respectively. When drug resistance was defined as intermediate and high levels, the concordance of the drug resistance profile of paired RNA and DNA for treatment-naïve patients was up to 97.0% (64/66), while the concordance for treatment-experienced patients was only 80.8% (63/78).

**Table 4:** Distribution of major DRMs in HIV RNA in treatment-experienced patients with discrepancies in DRMs between plasma RNA and PBMC DNA and drug resistance analysis.

Sample code	Subtype	RT		PR
		NRTI	NNRTI	PI
HIVHN2020-721RNA	B	M41L,D67G,K70R,L74I,V75T, M184V,T215F,K219E	Y181C	
HIVHN2020-750RNA	B	M184V	**K103N**	
HIVHN2020-839RNA	B		K103N	**I54IV, V82VA**
HIVHN2020-865RNA	B	**K65R**	K103N, **Y181C**	
HIVHN2020-881RNA	B	M41L,E44A,T69D,V75M,F77L, M184V,L210W,T215Y	A98G, K103H, V108I, Y181C, G190A	M46I,I47V,I54V,V82F,L33F,F53L
HIVHN2020-950RNA	CRF01_AE	A62V, K65R, M184I	K101H,V106I,Y181C,G190S	
HIVHN2020-954RNA	CRF01_AE	K65R, M184V	K101E, Y181C, G190S	
HIVHN2020-983RNA	B	K65R,**Y115F,M184V**,K219E	**L100I, K103NS, V106VI**	
HIVHN2020-990RNA	CRF01_AE	**L74LI, M184V**	**K103N**, V108I, V179E, **H221HY**	
HIVHN2021-2RNA	CRF01_AE	D67N, K70E, M184I, K219R	V108I, E138A, V179L, H221Y	
HIVHN2021-26RNA	B	D67DN, **K70KR**, M184V, **K219Q**	K103N, P225H	
HIVHN2021-40RNA	CRF07_BC	K65R	Y181C	
HIVHN2021-104RNA	B	**M41ML**,D67N,K70R,M184V, T215FY,**K219Q**	A98G, K101E, **V108VI**, G190A	
HIVHN2021-105RNA	CRF01_AE	**K70KQ, M184V**	**V106M**, V179D	
HIVHN2021-112RNA	CRF55_01B	M41L, L74I, M184V	A98G, K103N, V179E, P225H	
HIVHN2021-113RNA	B	**L74I**, M184V		**M46I, I54V, V82F**
HIVHN2021-114RNA	CRF01_AE	K65R, L74V, Y115F	G190Q, H221Y	
HIVHN2021-118RNA	B	K65R, Y115F, M184V		M46I, I47V, I54V, V82F, K43T, F53FL
HIVHN2021-119RNA	B	M41L, E44ED, M184V, L210W, T215Y, K219N	**K101H**, K103N, V108I, Y181C	
HIVHN2021-337RNA	B	**K65R, M184V**	**K101HQ**, V106I, Y181C, G190S, **H221HY**	
HIVHN2021-341RNA	CRF01_AE	K65R, M184V	K103NS	

Discordant DRMs between plasma RNA and PBMC DNA are shown in bold.

**Table 5:** Distribution of major DRMs in HIV proviral DNA in treatment-experienced patients with discrepancies in DRMs between plasma RNA and PBMC DNA and drug resistance analysis.

Sample code	Subtype	RT		PR
		NRTI	NNRTI	PI
HIVHN2020-721DNA	B	M41L, D67DG, K70R, L74LI, V75VAIT, M184V, T215FI, K219E	**K103N**, Y181C	**M46MI,I54IV,V82VF,** **I84IV, K43KT**
HIVHN2020-750DNA	B	M184V		
HIVHN2020-839DNA	B	**M184MV**	K103N	
HIVHN2020-865DNA	B		K103N	
HIVHN2020-881DNA	B	M41L,E44A,T69D,**K70KR**,V75VIM, F77FL,M184MIV,L210W,T215Y	A98AG, K103H, V108I, Y181C, G190A	M46MI,I47IV,I54IV,V82F, L33LF,F53FL
HIVHN2020-950DNA	CRF01_AE	A62AV, K65KR, M184I	K101H, V106I, Y181C, G190S, **M230MI**	
HIVHN2020-954DNA	CRF01_AE	K65R, M184MV	K101E,Y181C,G190S,**H221HY**	
HIVHN2020-983DNA	B	K65R, K219E		
HIVHN2020-990DNA	CRF01_AE		V108I, V179E	
HIVHN2021-2DNA	CRF01_AE	D67N, K70E, M184I, K219R	V108I, E138A, V179L, H221Y, **K238RT**	
HIVHN2021-26DNA	B	D67DN, M184V	K103N, P225H	
HIVHN2021-40DNA	CRF07_BC	K65R, **M184MI**	**K101KE, K103KN**, Y181C, **G190GRS**	
HIVHN2021-104DNA	B	D67DN, K70KR, M184MV, T215TFIS	A98AG, K101KE, G190AV	
HIVHN2021-105DNA	CRF01_AE		V179DE	
HIVHN2021-112DNA	CRF55_01B	M41L, L74I, M184V, **K219KR**	A98G, K103N, V179E, P225H	**K20KIT**
HIVHN2021-113DNA	B	M184MV	**K101KE, V106VI, V108VI, Y181YC, G190A**	
HIVHN2021-114DNA	CRF01_AE	K65KR, L74LV, Y115YF	**G190GEQR**, H221HY	
HIVHN2021-118DNA	B	K65R, Y115F, M184V	**Y181YC**	M46I, I47V, I54V, V82F, K43T, F53FL
HIVHN2021-119DNA	B	M41L, E44ED, M184V, L210W, T215Y, K219N	K103N, V108I, Y181C	
HIVHN2021-337DNA	B	**L74LV**	V106I, **V108VI**, Y181YC, G190GS	
HIVHN2021-341DNA	CRF01_AE	K65R, M184V	**L100LI**, K103NS	

Discordant DRMs between plasma RNA and PBMC DNA are shown in bold.

**Table 6:** Analysis of HIV RNA-based drug resistance in treatment-experienced patients with discrepancies in DRMs between plasma RNA and PBMC DNA.

Sample code	NRTIs							NNRTIs					PIs							
	ABC	AZT	D4T	DDI	FTC	3TC	TDF	DOR	EFV	ETR	NVP	RPV	ATV/r	DRV/r	FPV/r	IDV/r	LPV/r	NFV	SQV/r	TPV/r
HIVHN2020-721RNA	H	H	H	H	H	H	H	**S**	I	I	H	I	**S**	**S**	**S**	**S**	**S**	**S**	**S**	**S**
HIVHN2020-750RNA	L	S	S	S	H	H	S	S	**H**	S	**H**	S	S	S	S	S	S	S	S	S
HIVHN2020-839RNA	**S**	S	S	S	**S**	**S**	S	S	H	S	H	S	S	S	**I**	**I**	**I**	**H**	**I**	**L**
HIVHN2020-865RNA	**I**	S	**H**	**H**	**I**	**I**	**H**	**L**	H	**I**	H	**I**	S	S	S	S	S	S	S	S
HIVHN2020-881RNA	H	H	H	H	H	H	I	H	H	H	H	H	H	I	H	H	H	H	H	H
HIVHN2020-950RNA	H	S	I	H	H	H	I	H	H	H	H	H	S	S	S	S	S	S	S	S
HIVHN2020-954RNA	H	S	I	H	H	H	I	H	H	H	H	H	S	S	S	S	S	S	S	S
HIVHN2020-983RNA	H	S	H	H	H	H	H	**I**	**H**	**I**	**H**	**H**	S	S	S	S	S	S	S	S
HIVHN2020-990RNA	**H**	S	S	**H**	**H**	**H**	S	I	H	**L**	H	**L**	S	S	S	S	S	S	S	S
HIVHN2021-2RNA	I	S	I	I	H	H	L	I	I	I	I	I	S	S	S	S	S	S	S	S
HIVHN2021-26RNA	H	**I**	**I**	I	H	H	**L**	I	H	S	H	S	S	S	S	S	S	S	S	S
HIVHN2021-40RNA	I	S	H	H	I	I	H	**S**	I	I	H	I	S	S	S	S	S	S	S	S
HIVHN2021-104RNA	H	**H**	**H**	H	H	H	**I**	I	H	I	H	H	S	S	S	S	S	S	S	S
HIVHN2021-105RNA	**I**	S	**L**	**L**	**H**	**H**	**L**	**I**	**H**	S	**H**	**L**	S	S	S	S	S	S	S	S
HIVHN2021-112RNA	H	**S**	**S**	H	H	H	S	I	H	L	H	L	S	S	S	S	S	**S**	S	S
HIVHN2021-113RNA	H	S	S	**H**	H	H	S	**S**	**S**	**S**	**S**	**S**	**H**	**L**	**H**	**H**	**H**	**H**	**I**	**L**
HIVHN2021-114RNA	H	S	H	H	I	I	H	I	H	I	H	H	S	S	S	S	S	S	S	S
HIVHN2021-118RNA	H	S	I	H	H	H	H	S	**S**	**S**	**S**	**S**	H	L	H	H	H	H	H	H
HIVHN2021-119RNA	H	H	H	H	H	H	I	I	H	I	H	I	S	S	S	S	S	S	S	S
HIVHN2021-337RNA	H	S	**I**	H	**H**	**H**	**I**	H	H	H	H	H	S	S	S	S	S	S	S	S
HIVHN2021-341RNA	H	S	I	H	H	H	I	**S**	H	**S**	H	**S**	S	S	S	S	S	S	S	S

ABC, abacavir; AZT, zidovudine; D4T, stavudine; DDI, didanosine; FTC, emtricitabine; 3TC, lamivudine; TDF, tenofovir; DOR, doravirine; EFV, efavirenz; ETR, etravirine; NVP, nevirapine; RPV, rilpivirine; ATV/r, atazanavir/r; DRV/r, darunavir/r; FPV/r, fosamprenavir/r; IDV/r, indinavir/r; LPV/r, lopinavir/r; NFV, nelfinavir (NFV); SQV/r, saquinavir/r; TPV/r, tipranavir/r. Discordant DR between plasma RNA and PBMC DNA were shown in bold.

**Table 7:** Analysis of HIV proviral DNA-based drug resistance in treatment-experienced patients with discrepancies in DRMs between plasma RNA and PBMC DNA.

Sample code	NRTIs							NNRTIs					PIs							
	ABC	AZT	D4T	DDI	FTC	3TC	TDF	DOR	EFV	ETR	NVP	RPV	ATV/r	DRV/r	FPV/r	IDV/r	LPV/r	NFV	SQV/r	TPV/r
HIVHN2020-721DNA	H	H	H	H	H	H	H	**L**	H	I	H	I	**H**	**I**	**H**	**H**	**H**	**H**	**H**	**H**
HIVHN2020-750DNA	L	S	S	S	H	H	S	S	**S**	S	**S**	S	S	S	S	S	S	S	S	S
HIVHN2020-839DNA	**L**	**S**	S	S	**H**	**H**	S	S	H	S	H	S	S	S	**S**	**S**	**S**	**S**	**S**	**S**
HIVHN2020-865DNA	**S**	S	**S**	**S**	**S**	**S**	**S**	**S**	H	**S**	H	**S**	S	S	S	S	S	S	S	S
HIVHN2020-881DNA	H	H	H	H	H	H	H	H	H	H	H	H	H	I	H	H	H	H	H	H
HIVHN2020-950DNA	H	S	I	H	H	H	I	H	H	H	H	H	S	S	S	S	S	S	S	S
HIVHN2020-954DNA	H	S	I	H	H	H	I	H	H	H	H	H	S	S	S	S	S	S	S	S
HIVHN2020-983DNA	I	S	H	H	I	I	H	**S**	**S**	**S**	**S**	**S**	S	S	S	S	S	S	S	S
HIVHN2020-990DNA	**S**	S	S	**S**	**S**	**S**	S	L	L	**S**	L	**S**	S	S	S	S	S	S	S	S
HIVHN2021-2DNA	I	S	I	I	H	H	L	I	H	I	H	I	S	S	S	S	S	S	S	S
HIVHN2021-26DNA	L	**S**	**S**	L	H	H	**S**	I	H	S	H	S	S	S	S	S	S	S	S	S
HIVHN2021-40DNA	H	S	I	H	H	H	I	**H**	H	H	H	H	S	S	S	S	S	S	S	S
HIVHN2021-104DNA	L	**S**	**S**	L	H	H	**S**	L	H	L	H	I	S	S	S	S	S	S	S	S
HIVHN2021-105DNA	**S**	S	**S**	**S**	**S**	**S**	**S**	**S**	**S**	S	**S**	**S**	S	S	S	S	S	S	S	S
HIVHN2021-112DNA	H	**L**	**L**	H	H	H	S	I	H	L	H	L	S	S	S	S	S	**L**	S	S
HIVHN2021-113DNA	L	S	S	**S**	H	H	S	**H**	**H**	**H**	**H**	**H**	**S**	**S**	**S**	**S**	**S**	**S**	**S**	**S**
HIVHN2021-114DNA	H	S	H	H	I	I	H	H	H	I	H	H	S	S	S	S	S	S	S	S
HIVHN2021-118DNA	H	S	I	H	H	H	H	S	**I**	**I**	**H**	**I**	H	L	H	H	H	H	H	H
HIVHN2021-119DNA	H	H	H	H	H	H	I	I	H	I	H	I	S	S	S	S	S	S	S	S
HIVHN2021-337DNA	I	S	**S**	H	**S**	**S**	**S**	H	H	H	H	H	S	S	S	S	S	S	S	S
HIVHN2021-341DNA	H	S	I	H	H	H	I	**I**	H	**I**	H	**H**	S	S	S	S	S	S	S	S

ABC, abacavir; AZT, zidovudine; D4T, stavudine; DDI, didanosine; FTC, emtricitabine; 3TC, lamivudine; TDF, tenofovir; DOR, doravirine; EFV, efavirenz; ETR, etravirine; NVP, nevirapine; RPV, rilpivirine; ATV/r, atazanavir/r; DRV/r, darunavir/r; FPV/r, fosamprenavir/r; IDV/r, indinavir/r; LPV/r, lopinavir/r; NFV, nelfinavir (NFV); SQV/r, saquinavir/r; TPV/r, tipranavir/r. Discordant DR between plasma RNA and PBMC DNA were shown in bold.

### Paired plasma RNA and PBMC DNA sequences clustered together in most cases

**Figure 1:**
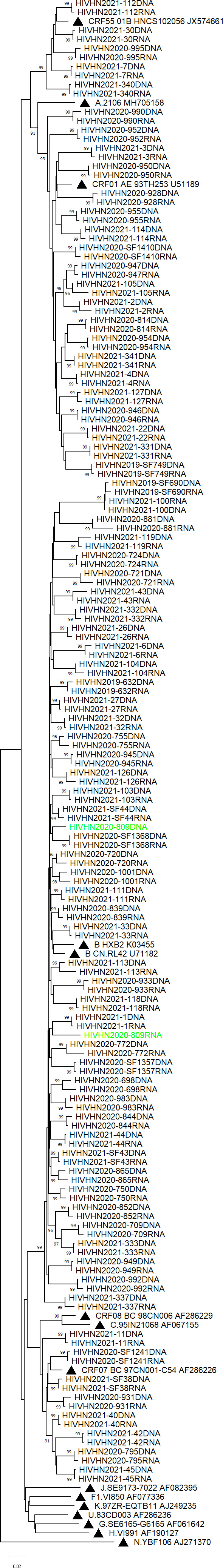
Phylogenetic tree based on pol sequences derived from treatment-naive patients. The tree was constructed using Molecular Evolution-ary Genetic Analysis (MEGA) software (version X) based on the neighbor-joining method and the Tamura-Nei model with 1000 bootstrap replicates. Bootstrap values higher than 85% are shown at the corresponding nodes.

**Figure 2:**
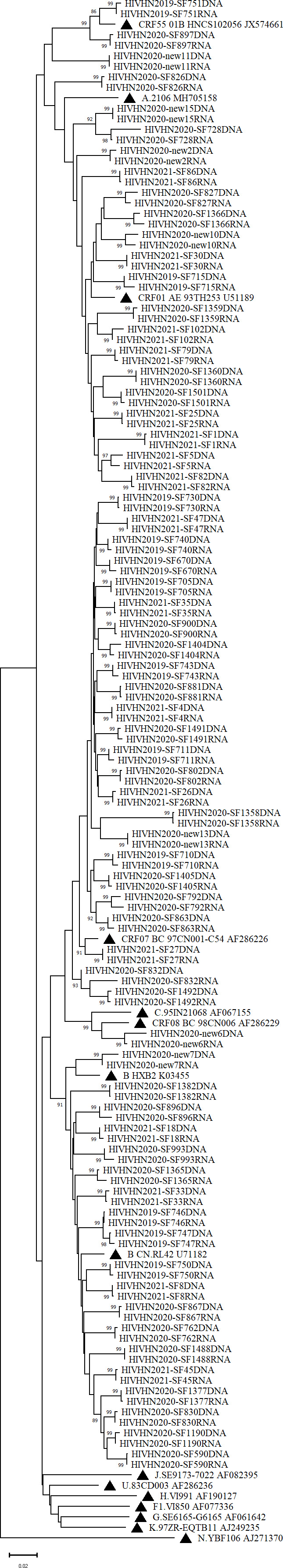
Phylogenetic tree based on pol sequences derived from treated patients. The tree was constructed using Molecular Evolutionary Genetic Analysis (MEGA) software (version X) based on the neighbor-joining method and the Tamura-Nei model with1000 bootstrap replicates. Bootstrap values higher than 85% were shown at the corresponding nodes.

Phylogenetic analysis based on the partial *pol* gene showed clusters of paired sequences derived from plasma RNA and PBMC DNA in most cases (**Figures 1**, **2**). However, paired RNA and DNA sequences clustered at different branches in one treatment-experienced patient (**Figure 2**).

## Discussion

Henan Province, located in eastern central China, has a population of more than 100 million residents. It was the earliest province afflicted with HIV in the 1990s due to transmission via illegal blood donation. Nearly 65,896 patients were diagnosed with HIV/AIDS in Henan Province by the end of October 2020, with an annual increase of more than 4,000, according to the Provincial Center for Disease Control and Prevention. Many factors, including lack of effective drugs, poverty, severe discrimination, and stigma resulted in high mortality in the early years. Since the emergency of HAART, AIDS has become a manageable chronic disease. Currently, all patients with HIV have freely access to ART in China. Free tests including annual CD4+ T cell count and VL, as well as genotypic drug resistance testing during failed ART are provided. However, a number of patients do not adhere to the prescribed medications and show high VL during annual routine VL test. These patients are then recommended to undergo genotypic drug resistance testing by physicians who are unaware of their poor adherence. The delay between recommendation and resampling for drug resistance testing can range from days to weeks. Most patients resume their medication or strictly follow the treatment regimen during this period, thereby leading to low plasma VL during resampling. To address this problem, we investigated the differences in DRMs between plasma HIV RNA and PBMC DNA in treatment-naïve and treatment-experienced patients, in an effort to determine the feasibility of PBMC DNA-based drug resistance testing.

Of the 66 treatment-naïve patients, DRMs were identified in 17 DNA and 14 RNA samples. The RNA and DNA samples showed 12 DRMs consistently, whereas 6 DRMs were inconsistent. Proviral DNA sequencing may overestimate DRMs as a result of hypermutations (Li et al., 2021). A few polymorphic mutations alone cannot lead to drug resistance. Two polymorphic mutations (V106I and V179D) were frequently found in treatment-naïve patients in this study. DNA sequencing revealed resistance mutations (E138A and M230I) not detected via RNA genotypic resistance testing. E138A mutation, which causes low-level resistance to rilpivirine, is also a polymorphic change depending on subtype and occurs in 0.5-5% of treatment-naïve patients (Huruy et al., 2018). M230I mutation often occurs as a result of APOBEC3 editing (Kieffer et al., 2005; Mulder et al., 2008). When drug resistance was defined as intermediate and high, up to 97% concordance existed between drug resistance in viral RNA and PBMC DNA, indicating that Sanger sequencing testing of plasma RNA or PBMC DNA may be interchangeable in treatment-naïve patients, similar to other countries or regions reported in previous studies (Vicenti et al., 2007; Demetriou et al., 2010). These treatment-naïve patients with intermediate- and/or high-level of resistance might be due to transmitted DRMs.

Consistent and inconsistent DRMs were observed in 42 and 21 matched RNA and DNA samples of treatment-experienced patients, respectively. In case of inconsistent DRMs, the extra DRMs in either plasma RNA or PBMC DNA generally altered the drug resistance profiles. The difference in drug resistance between paired RNA and DNA sequences in treatment-experienced patients might be attributed to the enrichment of drug-resistant strains in plasma during therapy failure, which can be easily identified compared with those in PBMCs (Delaugerre et al., 2012). The HIV DRM patterns detected in plasma RNA do not necessarily reflect those found in PBMC DNA (Turriziani et al., 2007). It has been suggested that the emergence of DRMs in PBMC proviruses was one year slower than that in plasma viruses (Bi et al., 2003). In another study, all resistance mutations associated with prior virological failure were not archived in the proviral DNA or decreased to a level below the threshold of detection. Therefore, testing for DNA genotypic resistance is less informative than testing for RNA genotypic resistance in patients with at least one prior virological failure (Lambert-Niclot et al., 2016). DRMs in PBMC DNA that are not found in plasma RNA represent archival resistance, which may be transmitted between individuals (Banks et al., 2012). In case of patients with ART failure, plasma genotypic testing combined with treatment history provides additional information than PBMC DNA genotypic resistance test alone for the assessment of the risk of virological failure after restarting therapy (Imaz et al., 2012).

One out of 144 paired samples was not tightly clustered, suggesting that circulating strains produced in the plasma may be derived from sources other than PBMCs (Chew et al., 2005; Banks et al., 2012; Huruy et al., 2018). In our study, some patients failing first-line ART showed no DRMs. Several studies have indicated that the latent reservoir is composed of a mixture of wild-type and drug-resistant strains (Turriziani et al., 2010). Thus, the failure of HAART in some patients cannot be solely attributed to drug resistance. Other reasons for treatment failure include poor adherence due to adverse effects, host factors or pharmacokinetic failure, which also need to be evaluated by clinicians (Chew et al., 2005). It has been suggested that transcriptionally active proviruses evolve dynamically under selection pressure by host factors (Einkauf et al., 2022).

The present study has several limitations. First, detailed information regarding patients’ adherence was not available, which hindered in-depth analysis of the different DRMs between matched RNA and DNA samples. Theoretically, plasma RNA changes dynamically and reflects the constant emergence of drug-resistant variants, while the viral DNA shows rather stable dynamics. Second, low-level DRMs (< 20%) are not detectable by the conventional Sanger sequencing, suggesting the need for more sensitive techniques, such as ultra-deep sequencing or next generation sequencing to evaluate the presence of DRMs. Third, drug resistance to integrase strand transfer inhibitors (INSTIs) was not determined. However, due to the low prevalence of resistance to INSTIs and the small sample size, a significant difference might not be found in this study.

Our data support that genotypic drug resistance testing based on plasma RNA or PBMC DNA may be interchangeable in treatment-naïve patients and resistance testing based on PBMC DNA can be used as an alternative in treatment-experienced patients failing ART. However, the results should be interpreted with caution and additional studies should be conducted in the future.

## Ethical approval

This study was approved by the Institutional Ethics Committee of The Sixth People’s Hospital of Zhengzhou, China. Informed consent Signed informed consent was obtained from each patient before the collection of blood samples.

## Data availability statement

The original contributions presented in the study are included in the article/**Supplementary Materials**. Further inquiries can be directed to the corresponding authors.

## Ethics statement

The studies involving humans were approved by ethics committee of the Sixth People’s Hospital of Zhengzhou. The studies were conducted in accordance with the local legislation and institutional requirements. The participants provided their written informed consent to participate in this study.

## Author contributions

YH and YS: Conceptualization, Investigation, Project administration, Funding acquisition. JM, ZC, JL, SW and XC, XY: Methodology, Formal analysis, Data curation, Visualization. YH, QZ and CF: Supervision, Resources, Review & editing. JM: Writing - original draft. All authors contributed to the article and approved the submitted version.
