# Supplementary material for: Consistency of drug-resistant mutations in plasma and peripheral blood mononuclear cells of patients with treatment-naïve and treatment-experienced HIV-1 infection
Source: Front Cell Infect Microbiol. 2023 Dec 19;13:1249837. doi: 10.3389/fcimb.2023.1249837 (PMC10766352; doi:10.3389/fcimb.2023.1249837)
Supplement: Supplementary file 1 [file Presentation_1.pptx]

## Slide 1
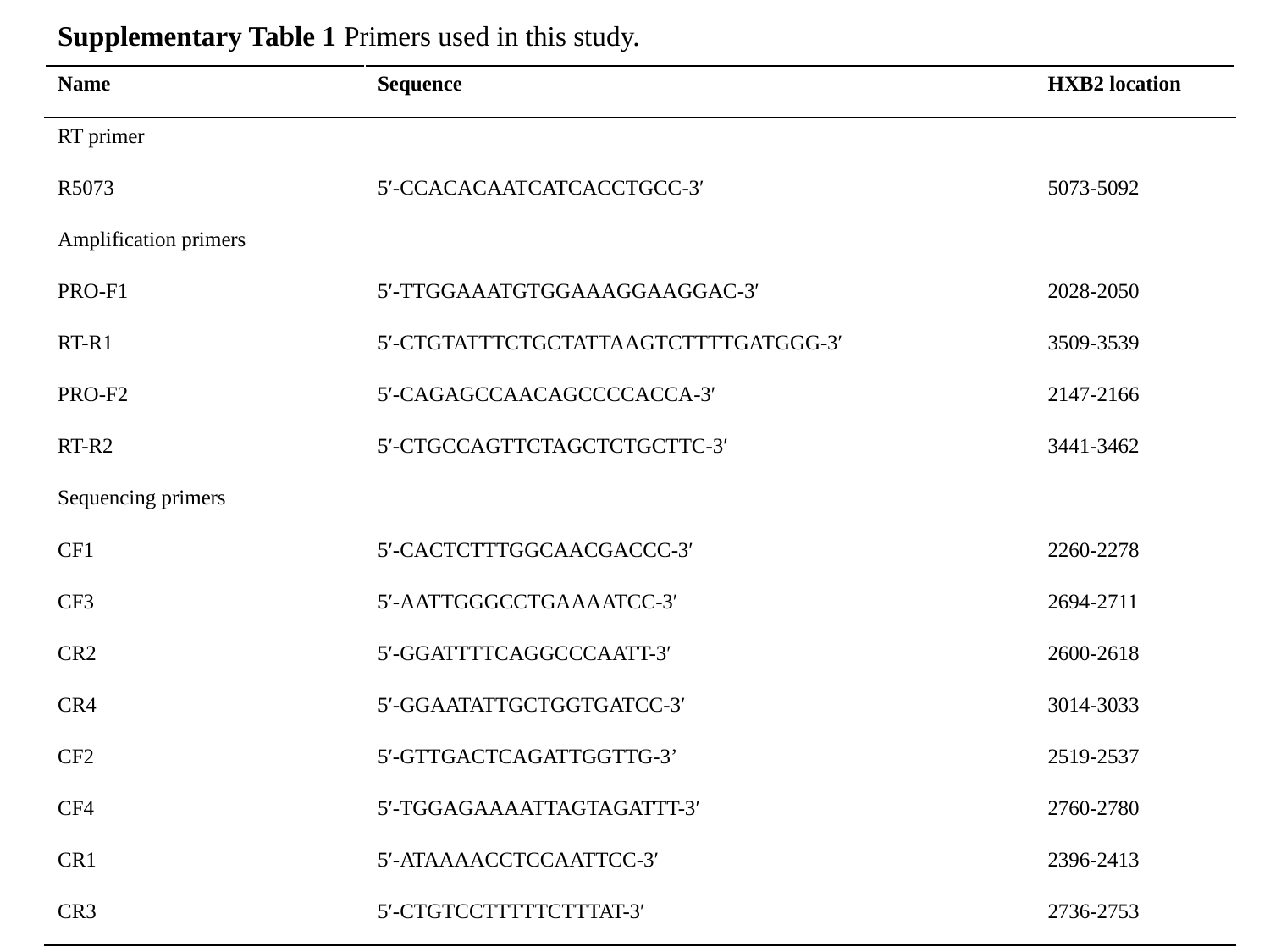

# Supplementary Table 1 Primers used in this study.
| Name | Sequence | HXB2 location |
| --- | --- | --- |
| RT primer | | |
| R5073 | 5′-CCACACAATCATCACCTGCC-3′ | 5073-5092 |
| Amplification primers | | |
| PRO-F1 | 5′-TTGGAAATGTGGAAAGGAAGGAC-3′ | 2028-2050 |
| RT-R1 | 5′-CTGTATTTCTGCTATTAAGTCTTTTGATGGG-3′ | 3509-3539 |
| PRO-F2 | 5′-CAGAGCCAACAGCCCCACCA-3′ | 2147-2166 |
| RT-R2 | 5′-CTGCCAGTTCTAGCTCTGCTTC-3′ | 3441-3462 |
| Sequencing primers | | |
| CF1 | 5′-CACTCTTTGGCAACGACCC-3′ | 2260-2278 |
| CF3 | 5′-AATTGGGCCTGAAAATCC-3′ | 2694-2711 |
| CR2 | 5′-GGATTTTCAGGCCCAATT-3′ | 2600-2618 |
| CR4 | 5′-GGAATATTGCTGGTGATCC-3′ | 3014-3033 |
| CF2 | 5′-GTTGACTCAGATTGGTTG-3’ | 2519-2537 |
| CF4 | 5′-TGGAGAAAATTAGTAGATTT-3′ | 2760-2780 |
| CR1 | 5′-ATAAAACCTCCAATTCC-3′ | 2396-2413 |
| CR3 | 5′-CTGTCCTTTTTCTTTAT-3′ | 2736-2753 |

## Slide 2
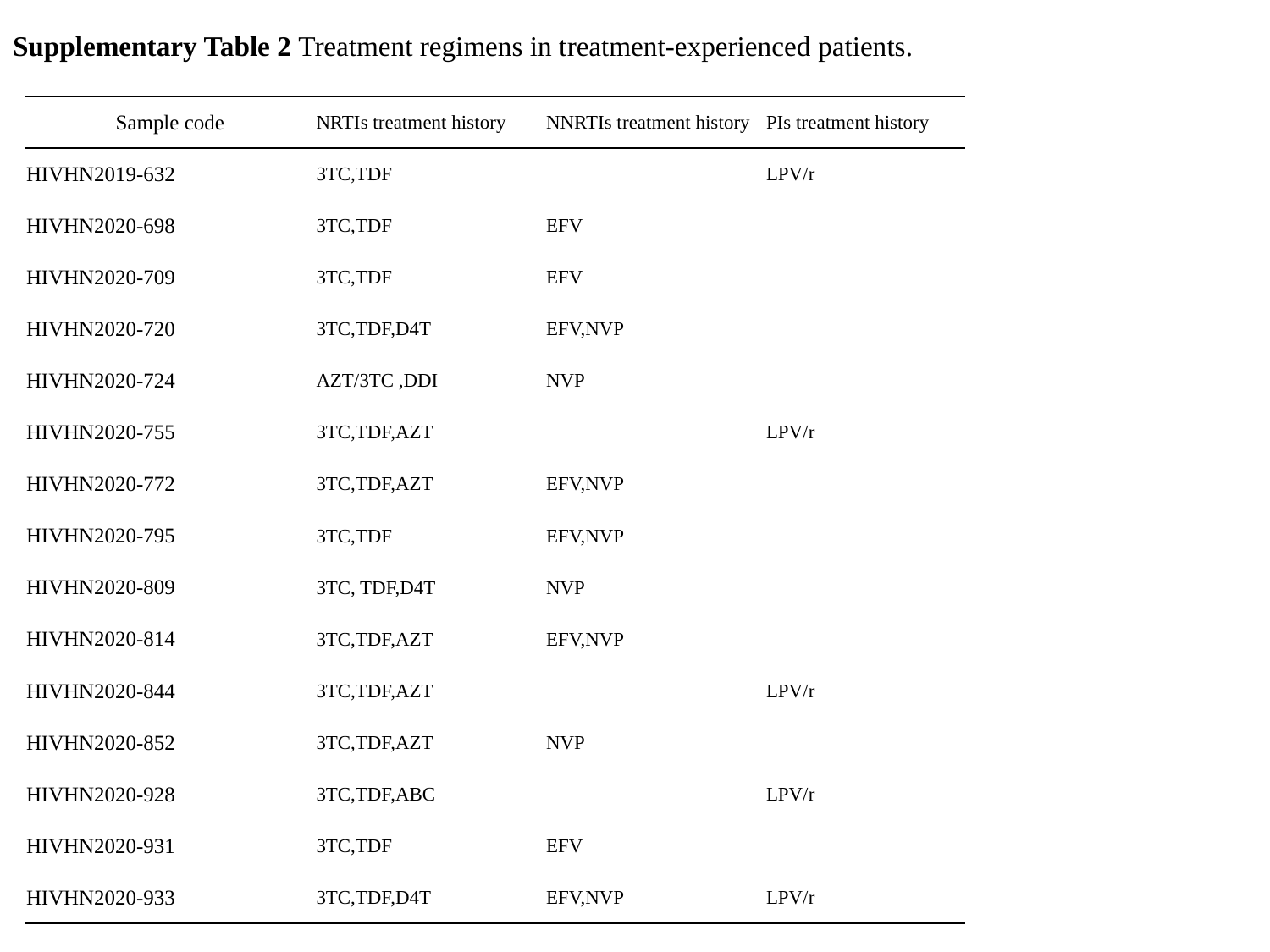

# Supplementary Table 2 Treatment regimens in treatment-experienced patients.
| Sample code | NRTIs treatment history | NNRTIs treatment history | PIs treatment history |
| --- | --- | --- | --- |
| HIVHN2019-632 | 3TC,TDF | | LPV/r |
| HIVHN2020-698 | 3TC,TDF | EFV | |
| HIVHN2020-709 | 3TC,TDF | EFV | |
| HIVHN2020-720 | 3TC,TDF,D4T | EFV,NVP | |
| HIVHN2020-724 | AZT/3TC ,DDI | NVP | |
| HIVHN2020-755 | 3TC,TDF,AZT | | LPV/r |
| HIVHN2020-772 | 3TC,TDF,AZT | EFV,NVP | |
| HIVHN2020-795 | 3TC,TDF | EFV,NVP | |
| HIVHN2020-809 | 3TC, TDF,D4T | NVP | |
| HIVHN2020-814 | 3TC,TDF,AZT | EFV,NVP | |
| HIVHN2020-844 | 3TC,TDF,AZT | | LPV/r |
| HIVHN2020-852 | 3TC,TDF,AZT | NVP | |
| HIVHN2020-928 | 3TC,TDF,ABC | | LPV/r |
| HIVHN2020-931 | 3TC,TDF | EFV | |
| HIVHN2020-933 | 3TC,TDF,D4T | EFV,NVP | LPV/r |

## Slide 3
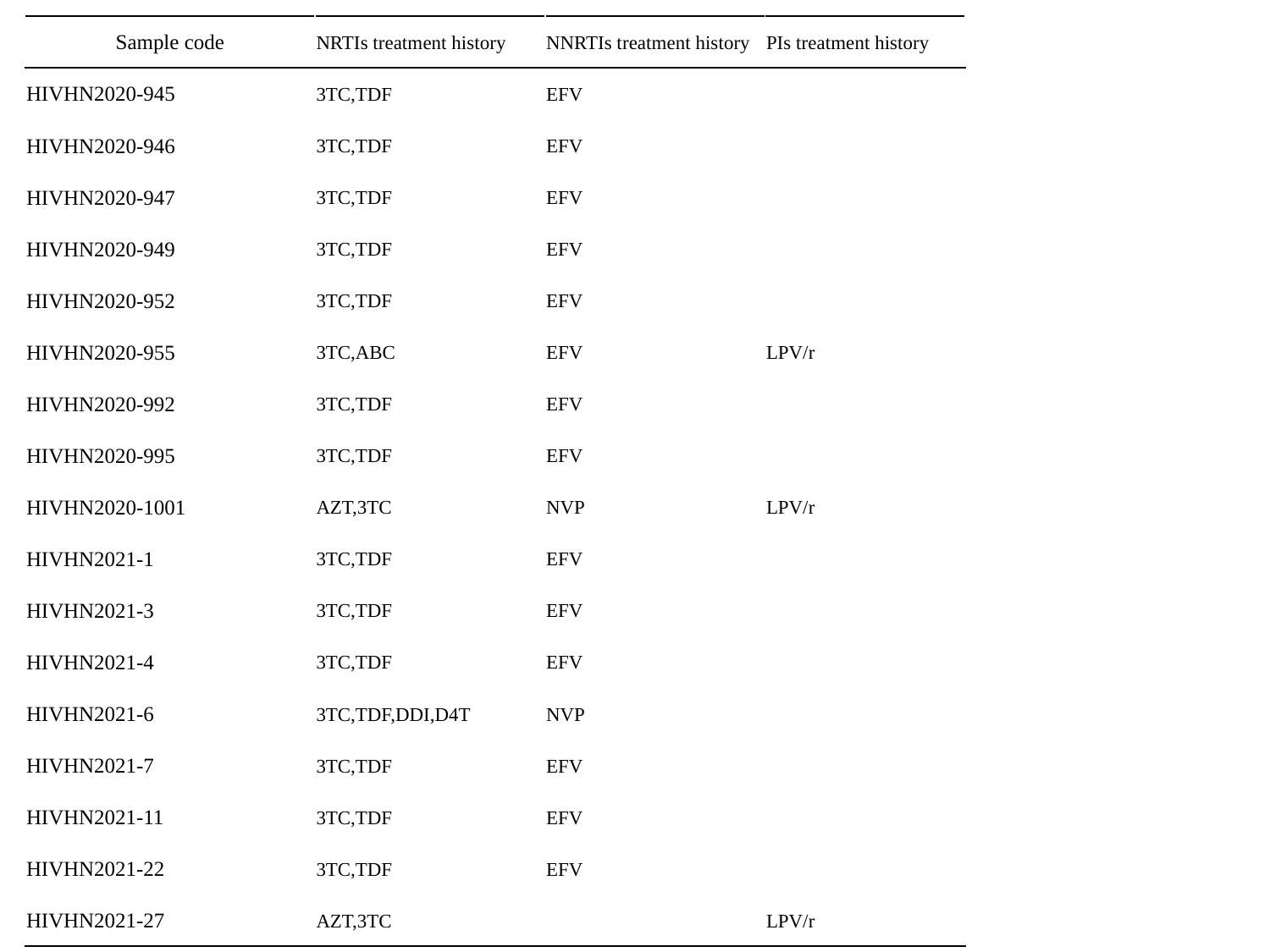

| Sample code | NRTIs treatment history | NNRTIs treatment history | PIs treatment history |
| --- | --- | --- | --- |
| HIVHN2020-945 | 3TC,TDF | EFV | |
| HIVHN2020-946 | 3TC,TDF | EFV | |
| HIVHN2020-947 | 3TC,TDF | EFV | |
| HIVHN2020-949 | 3TC,TDF | EFV | |
| HIVHN2020-952 | 3TC,TDF | EFV | |
| HIVHN2020-955 | 3TC,ABC | EFV | LPV/r |
| HIVHN2020-992 | 3TC,TDF | EFV | |
| HIVHN2020-995 | 3TC,TDF | EFV | |
| HIVHN2020-1001 | AZT,3TC | NVP | LPV/r |
| HIVHN2021-1 | 3TC,TDF | EFV | |
| HIVHN2021-3 | 3TC,TDF | EFV | |
| HIVHN2021-4 | 3TC,TDF | EFV | |
| HIVHN2021-6 | 3TC,TDF,DDI,D4T | NVP | |
| HIVHN2021-7 | 3TC,TDF | EFV | |
| HIVHN2021-11 | 3TC,TDF | EFV | |
| HIVHN2021-22 | 3TC,TDF | EFV | |
| HIVHN2021-27 | AZT,3TC | | LPV/r |

## Slide 4
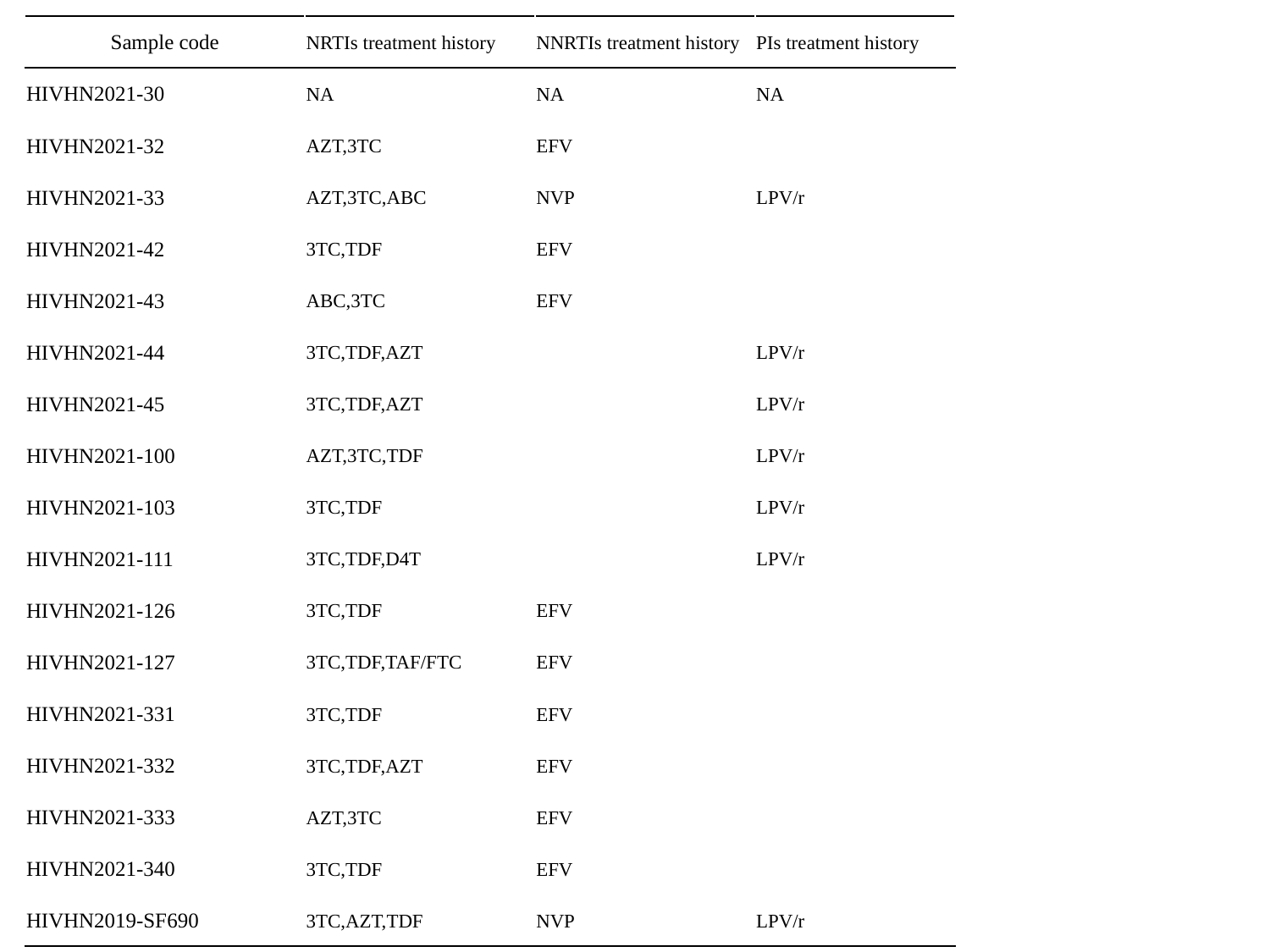

| Sample code | NRTIs treatment history | NNRTIs treatment history | PIs treatment history |
| --- | --- | --- | --- |
| HIVHN2021-30 | NA | NA | NA |
| HIVHN2021-32 | AZT,3TC | EFV | |
| HIVHN2021-33 | AZT,3TC,ABC | NVP | LPV/r |
| HIVHN2021-42 | 3TC,TDF | EFV | |
| HIVHN2021-43 | ABC,3TC | EFV | |
| HIVHN2021-44 | 3TC,TDF,AZT | | LPV/r |
| HIVHN2021-45 | 3TC,TDF,AZT | | LPV/r |
| HIVHN2021-100 | AZT,3TC,TDF | | LPV/r |
| HIVHN2021-103 | 3TC,TDF | | LPV/r |
| HIVHN2021-111 | 3TC,TDF,D4T | | LPV/r |
| HIVHN2021-126 | 3TC,TDF | EFV | |
| HIVHN2021-127 | 3TC,TDF,TAF/FTC | EFV | |
| HIVHN2021-331 | 3TC,TDF | EFV | |
| HIVHN2021-332 | 3TC,TDF,AZT | EFV | |
| HIVHN2021-333 | AZT,3TC | EFV | |
| HIVHN2021-340 | 3TC,TDF | EFV | |
| HIVHN2019-SF690 | 3TC,AZT,TDF | NVP | LPV/r |

## Slide 5
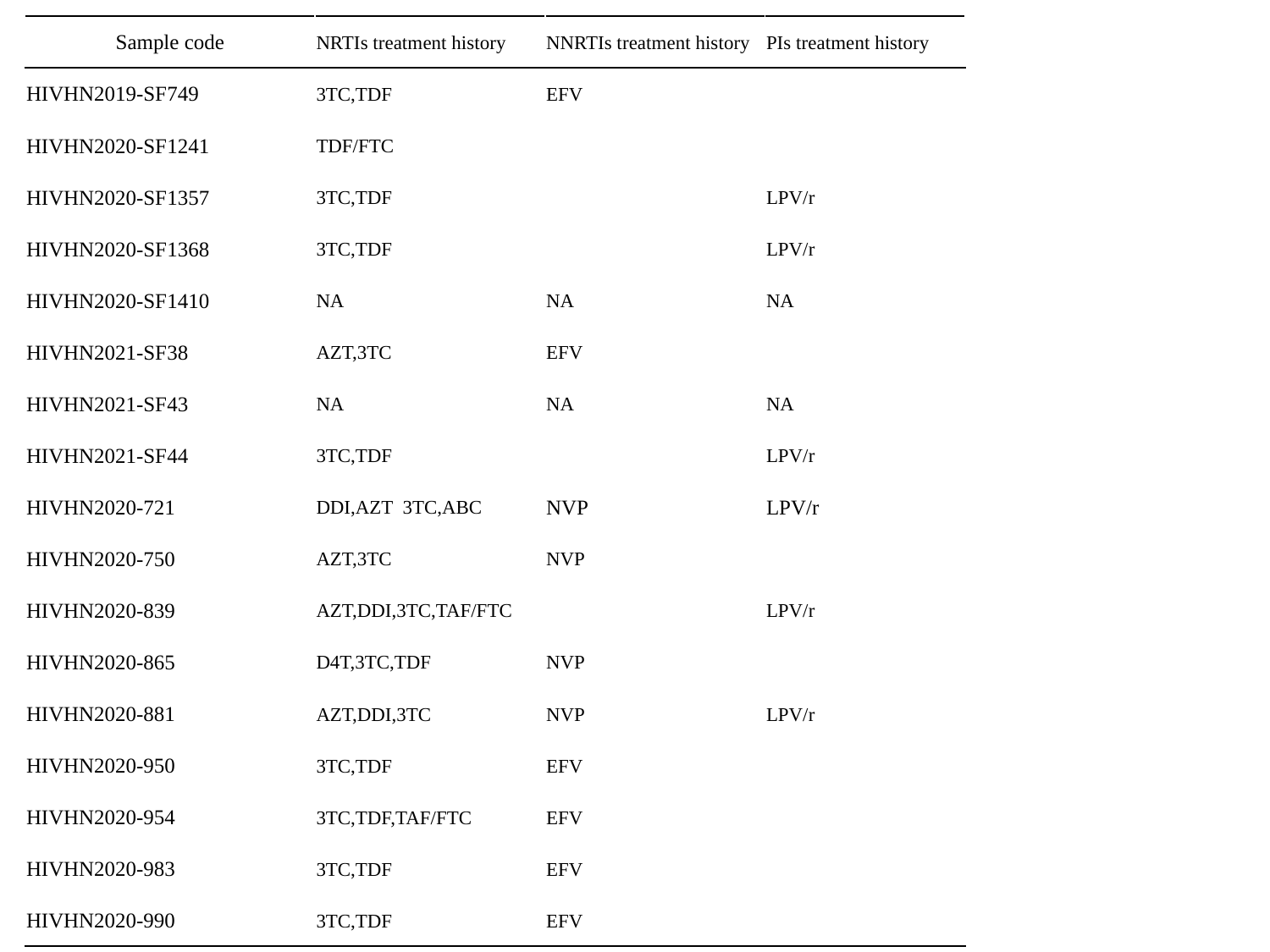

| Sample code | NRTIs treatment history | NNRTIs treatment history | PIs treatment history |
| --- | --- | --- | --- |
| HIVHN2019-SF749 | 3TC,TDF | EFV | |
| HIVHN2020-SF1241 | TDF/FTC | | |
| HIVHN2020-SF1357 | 3TC,TDF | | LPV/r |
| HIVHN2020-SF1368 | 3TC,TDF | | LPV/r |
| HIVHN2020-SF1410 | NA | NA | NA |
| HIVHN2021-SF38 | AZT,3TC | EFV | |
| HIVHN2021-SF43 | NA | NA | NA |
| HIVHN2021-SF44 | 3TC,TDF | | LPV/r |
| HIVHN2020-721 | DDI,AZT 3TC,ABC | NVP | LPV/r |
| HIVHN2020-750 | AZT,3TC | NVP | |
| HIVHN2020-839 | AZT,DDI,3TC,TAF/FTC | | LPV/r |
| HIVHN2020-865 | D4T,3TC,TDF | NVP | |
| HIVHN2020-881 | AZT,DDI,3TC | NVP | LPV/r |
| HIVHN2020-950 | 3TC,TDF | EFV | |
| HIVHN2020-954 | 3TC,TDF,TAF/FTC | EFV | |
| HIVHN2020-983 | 3TC,TDF | EFV | |
| HIVHN2020-990 | 3TC,TDF | EFV | |

## Slide 6
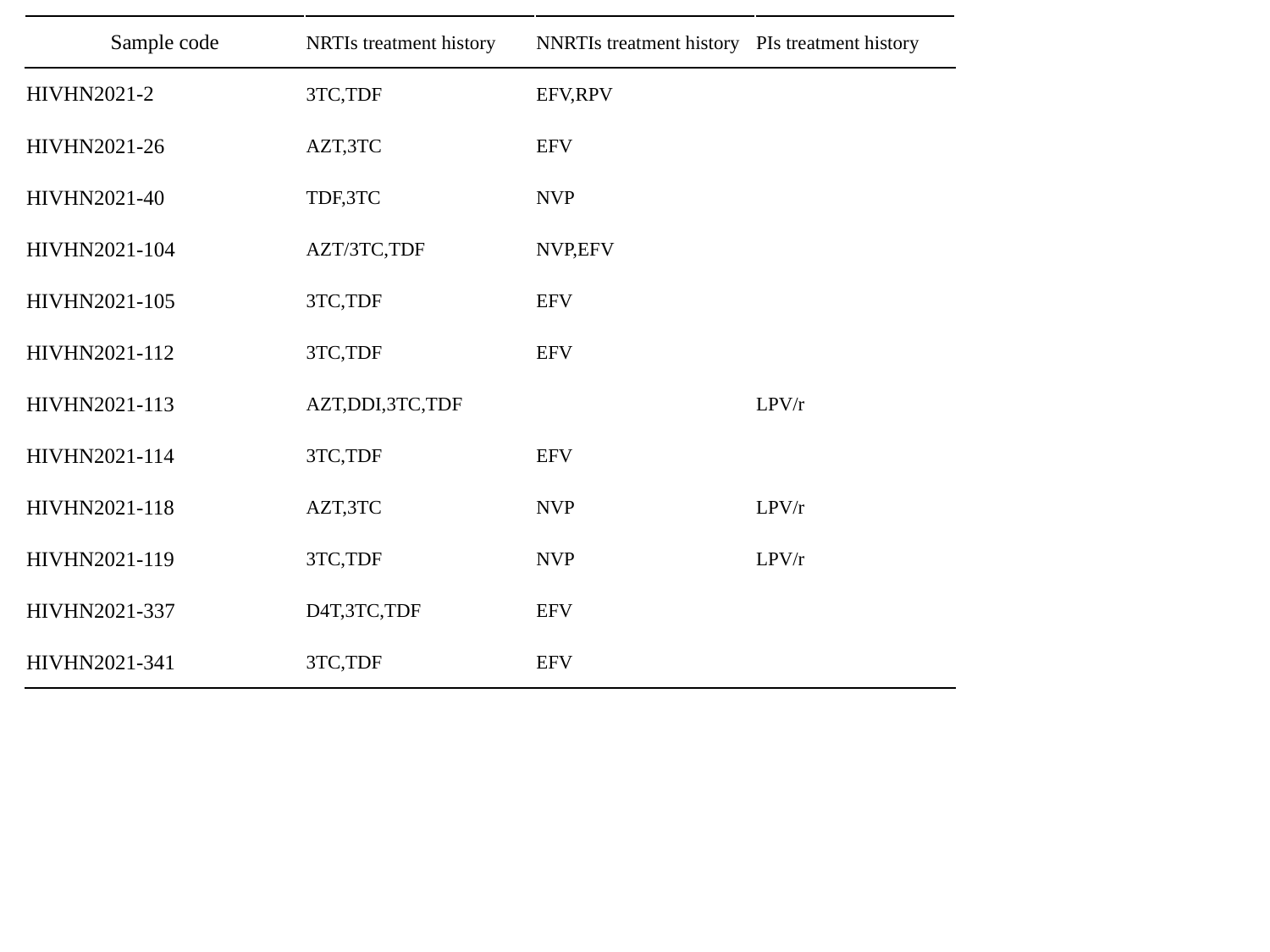

| Sample code | NRTIs treatment history | NNRTIs treatment history | PIs treatment history |
| --- | --- | --- | --- |
| HIVHN2021-2 | 3TC,TDF | EFV,RPV | |
| HIVHN2021-26 | AZT,3TC | EFV | |
| HIVHN2021-40 | TDF,3TC | NVP | |
| HIVHN2021-104 | AZT/3TC,TDF | NVP,EFV | |
| HIVHN2021-105 | 3TC,TDF | EFV | |
| HIVHN2021-112 | 3TC,TDF | EFV | |
| HIVHN2021-113 | AZT,DDI,3TC,TDF | | LPV/r |
| HIVHN2021-114 | 3TC,TDF | EFV | |
| HIVHN2021-118 | AZT,3TC | NVP | LPV/r |
| HIVHN2021-119 | 3TC,TDF | NVP | LPV/r |
| HIVHN2021-337 | D4T,3TC,TDF | EFV | |
| HIVHN2021-341 | 3TC,TDF | EFV | |
